# Pilot Nanostring PanCancer pathway analysis of colon adenocarcinoma in a tertiary healthcare centre in Kerala, India

**DOI:** 10.3332/ecancer.2021.1302

**Published:** 2021-10-12

**Authors:** Prasanth S Ariyannur, Reenu Anne Joy, Veena Menon, Roopa Rachel Paulose, Keechilat Pavithran, Damodaran M Vasudevan

**Affiliations:** 1 Department of Biochemistry and Molecular Biology, Amrita School of Medicine, Amrita Institute of Medical Sciences and Research Center, Amrita Vishwa Vidyapeetham, Kochi 682041, India; 2 Department of Molecular Biology, Amrita Institute of Medical Sciences and Research Center, Amrita Vishwa Vidyapeetham, Kochi 682041, India; 3 Department of Pathology, Amrita Institute of Medical Sciences and Research Center, Amrita Vishwa Vidyapeetham, Kochi 682041, India; 4 Department of Medical Oncology, Amrita Institute of Medical Sciences and Research Center, Amrita Vishwa Vidyapeetham, Kochi 682041, India; 5 Department of Health Sciences Research, Amrita Institute of Medical Sciences and Research Center, Amrita Vishwa Vidyapeetham, Kochi 682041, India

**Keywords:** colorectal cancer, south India, microsatellite instability, Nanostring, expression analysis, TCGA, GEPIA, TIMER

## Abstract

The prevalence of microsatellite instability and deoxyribonucleic acid mismatch repair deficiency in colorectal adenocarcinoma (CRC) cases is higher in India compared to western populations. No major study on the molecular pathogenesis is currently available in the Indian population. We conducted a pilot study to explore the differences in molecular pathogenesis of microsatellite stable (MSS) and microsatellite unstable CRC from a tertiary care centre in Kerala, South India. Using Nanostring PanCancer panel assay in Stage II colorectal adenocarcinoma, tumour tissues (*n* = 11) were compared against normal colon tissues (*n* = 4). Differentially expressed (DE) genes were identified and super-imposed onto colon adenocarcinoma cohort of The Cancer Genome Atlas (TCGA) data (TCGA Colon Adenocarcinoma (TCGA COAD)), from the Genome Expression Profiling Interactive Analysis and Tumor Immune Estimation Resource (TIMER) to compare the gene associations. Significant DE genes were 59 out of 730 (false discovery rate adj. *p*-value < 0.05), 18 of which had a fold-change |FC(log2)| ≥ 2. On superimposition to TCGA COAD, 33 genes were significant in both TCGA and current study. ETV4 was expressed significantly higher in MSS with no immune cell infiltration. Other significant DE genes with high FC(log2), unique to the study were INHBA, COL1A1, COL11A1, COMP, SFRP4 and SPP1, which were clustered in STRING network analysis and correlated with tumour-infiltrating immune cells in TIMER, suggesting a specific interaction pathway. The preliminary study suggests a distinct pathogenesis of MSS CRC involving ETV4 in the Indian population and warrants further clinically extensive and high-dimensional expression studies.

## Background

In the Western population, the molecular classification of colorectal cancers (CRCs) by consensus molecular subtyping, validated by The Cancer Genome Atlas (TCGA) Consortium studies [1, 2], classified CRC into the most common chromosomal instability [3, 4], followed by microsatellite instability due to defective deoxyribonucleic acid (DNA) mismatch repair (MMR) [1] constituting about one-fifth of cases, which comprises both sporadic and hereditary causes [5]. Less than 5% of CRC was found to be due to constitutional and inherited mutations in MMR genes – *MLH1*, *MSH2*, *MSH6*, *PMS2* and *EPCAM* [6]. The complexity of MMR function due to the involvement of more than 30 genes [7], epigenetic inactivation [8], microRNA mediated regulation of MMR gene transcripts [9],and hetero-oligomeric co-dependence of MMR proteins [10], leading to variable phenotypic penetrance are some of the very few known factors for diagnostic and scientific challenges of this syndrome.

Global molecular studies on CRC, such as TCGA, or genome-wide microsatellite instability analysis did not include representative cases from India [11]. The prevalence rate of CRC in India, averaging to 4.3 and 3.4 in 100,000 males and females, respectively, has been further increasing, as per epidemiological reports [12]. Pioneer studies in Southern India, conducted by a centre in the provincial state of Tamil Nadu, showed that 67% of cases had deficient MMR [13]. This was supported by higher prevalence rates in other studies from the state of Andhra Pradesh [14, 15]. In our population in the state of Kerala, deficient MMR was found in 27% of Stage II CRC [16]. These population studies show that the molecular pathogenesis of CRC in Indian population might be different from Western population.

To address this, we conducted a pilot study in a set of Stage II CRC from a tertiary cancer care centre in Kerala, India. The study compares tumours from both microsatellite unstable (MSI) and microsatellite stable (MSS) categories, with the histopathological features against multi-gene expression analysis using Nanostring Pan-Cancer pathway analysis. To identify unique expression signals in the population, the expression pattern of the gene signals obtained from local population were compared against the TGCA Colon Adenocarcinoma (TCGA COAD) data set using multiple tools such as Genome Expression Profiling Interactive Analysis (GEPIA) and Tumor Immune Estimation Resource (TIMER). Using these methods, we provide an approach to understand the difference in molecular pathogenesis in Indian CRC cases.

## Methods

### Samples

We selected 11 tumour tissues of Stage II CRC and 4 normal tissues for the comparative study using Nanostring nCounter analysis. Institutional scientific and ethics committee approval was obtained before the study. The histopathological features of tumour such as tumour type, differentiation, the extent of tumour invasion, lymphocyte infiltration, lympho-vascular and perineural invasion were assessed. Normal tissues from the adjacent normal regions of tumour samples were selected and 5 μm serial sections of formalin fixed paraffin embedded (FFPE) tissue blocks of normal and tumour were prepared. Tumour samples were obtained from subjects with an age of onset from late-30s through mid-70s. Eight samples were from the right/proximal colon (ileocecal and ascending colon), two from transverse colon and one from the sigmoid colon (Table 1 and Supplementary Dataset File S1). All the tumours were T_3_N_0_M_0_, according to the Union for International Cancer Control (UICC) staging. Six samples from the right colon had moderate lymphocyte infiltration (TIL). DNA MMR was assessed by immunohistochemical (IHC) reactivity to four standard MMR proteins (MLH1, MSH2, MSH6 & PMS2). These samples were confirmed by MSI-polymerase chain reaction using two mononucleotide repeat markers (BAT25, BAT26), and three quasi-monomorphic repeat markers (NR-21, NR-24, NR-27) [17]. Six tumour tissues had deficient MMR (MSI), and five had proficient MMR (MSS).

### Nanostring nCounter assay

The NanoString nCounter assay was conducted by a contract research organisation (Theracues Pvt Ltd, Bangalore, India), using validated commercial methods on a pay-by-service basis. Briefly, RNA was extracted from three-to-four 5 μm FFPE samples sections using commercial FFPE nucleic acid isolation kit (Roche Molecular Diagnostics), quantified and analysed for fragment distribution using a bioanalyzer (Agilent). About 140 ng of RNA was used for each probing assay. The fluorescence count obtained from the nCounter machine was normalised according to the positive controls and internal housekeeping reference genes. The housekeeping genes were selected using geNorm algorithm as per *NormqPCR* Bioconductor package. The median values of the probe set expression, after standardisation with standard expression sets, and clustering analysis was employed using appropriate package modules in R statistical program. The differentially expressed (DE) genes in the tumour were calculated by taking the geometric mean of the four normal tissues as the denominator and the geometric mean of all the tumour samples as the numerator. DE genes with false discovery rate (FDR) adjusted *p-*value < 0.05 were considered significant. The significant genes were used to generate principal component analysis (PCA) and heatmap using ClustVis webserver [18, 19]. Evaluation of differential expression values by group difference between microsatellite instability status, infiltration status as well as the anatomical location was done using Student *t*-test analysis separately for each gene, assuming unequal variance using *genefilter* Bioconductor package.

### Network analysis

In order to explore protein–protein interaction of the top DE genes, STRING database (string-db.org) [20], an integrated, publicly available resource, based on laboratory experiments in protein–protein interactions, conserved co-expression data, genomic context predictions and text mining from previous research, was used. The top 20 highly significant genes from the Nanostring expression analysis along with their log_2_ fold-change, FC(log_2_), values were entered into the protein-protein interaction network database (STRING.db) online tool to explore the possible protein–protein interactions among these top gene signals. The ranking of each of these proteins was determined from the FC values. The active interaction source parameters selected were *Text mining*, *Experiments*, *Databases*, *Co-expression*, *Neighborhood*, *Gene Fusion* and *Co-occurrence*. The meaning of network edges to depict the interaction lines was selected to choose distinct colours according to evidence available.

### Co-expression analysis

The significant DE genes identified were evaluated for co-expression analysis by GEPIA and TIMER databases. GEPIA and TIMER are online tools used to explore transcriptome data from the TCGA and genotype-tissue expression project projects [21, 22]. Using both databases, the correlation of each of the selected genes, found to be expressed together from the test samples, was analysed for their co-expression correlation coefficient in the TCGA COAD. The genes with correlation coefficient > 0.5 and adj. *p* < 0.05 were analysed further.

### Immune cell infiltrate analysis

TIMER is a web-based database tool to analyse tumour-infiltrating immune cells such as B Lymphocytes (B-cells), CD4^+^ T cells, CD8^+^ T cells, neutrophils, macrophages and dendritic cells from gene expression profiles of TCGA data [22]. The selected genes from the study samples were compared against the immune cell infiltrate in the tissues of TCGA COAD cohort, to correlate the type and abundance of infiltrating immune cells associated with the given set of unique genes identified in the local population. The correlation coefficient of each cell type in the TIMER database against the top enriched genes from the current study was analysed and clustered using hierarchical clustering analysis by *hclust*, and *dist* functions in the R program.

## Results

### Nanostring expression analysis

**Figure 1.**
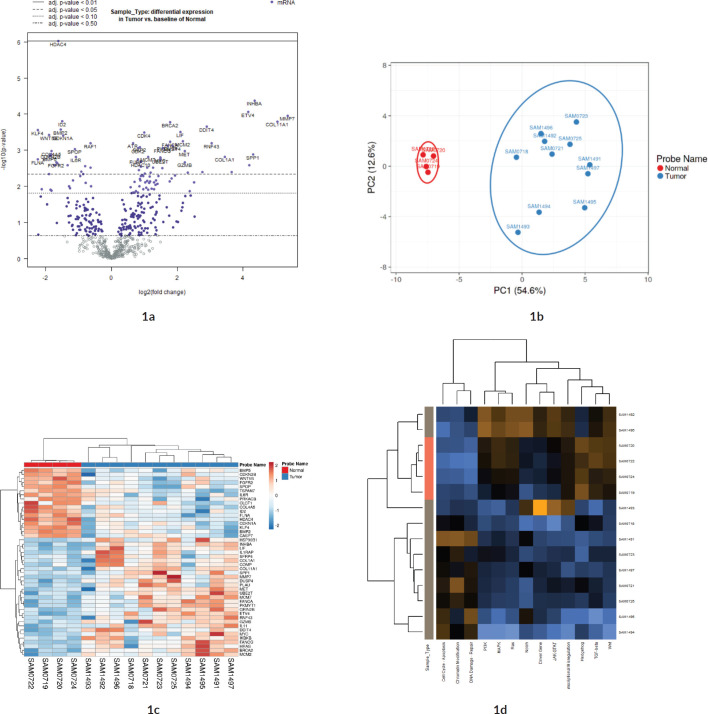
Differential expression analysis. (a): Volcano plot displaying the expression pattern of the entire gene set. Y-axis is −log_10_(p-value) and X-axis is log_2_(FC) of each of the covariates. Significant genes fall at the top of the plot above the horizontal lines, and highly DE genes fall to either side. Horizontal lines indicate various FDR thresholds or p-value thresholds if there is no adjustment to the p-values. Genes are coloured appropriately if the resulting p-value is below the given adj. p-value threshold. Fifty-nine genes that had FDR adj. p-value < 0.05 are shown in the plot. (b): Venn diagram showing PCA of FC of top 20 genes. (c): Heatmap showing the 46 DE genes with adj. p-value < 0.05 and |FC(log_2_)| > 1. The normal and tumour samples are coloured coded in the top margin. (d): Heatmap showing pathway score depicting an overview of how the pattern of pathway scores change across samples to understand how pathway scores cluster together and which samples exhibit similar pathway score profiles. Orange indicates high scores; blue indicates low scores. Scores are displayed on the same scale via a Z-transformation.

Out of the 622 DE genes from 730 target signals in the PanCancer Pathway set, 101 genes were found to have an FDR adjusted *p*-value < 0.1 and 59 genes were found to be significant (FDR adj. *p*-value < 0.05) across the samples (Figure 1a, Supplementary Dataset File S2). Of these, 16 genes were upregulated, (FC(log_2_) > 2) while 2 genes were downregulated (FC(log_2_) ≤ −2) (see Sheet 4 of Supplementary Dataset File S2). These genes are listed in Supplementary Dataset File S2 with their corresponding FDR adjusted *p*-values and FC(log_2_) values. PCA of FC top 20 significant genes is depicted in Figure 1b. FC values of 20 top significant genes were clustered among tumour samples, and the expression pattern was reversed in the normal tissues. With the Nanostring pathway analysis, signals associated with cell cycle and apoptosis, chromatin modification and DNA damage repair were shown to be upregulated in tumour samples and downregulated in normal tissues, with a few exceptions in certain samples (Figure 1e). FC and overall pathway score for the chromatin modification set and DNA damage repair in different samples did not correspond to their MMR status. This might be influenced by the tumour cells as well as TILs and macrophages, which was further explored by the GEPIA and TIMER correlation analyses.

### Group comparison

**Figure 2.**
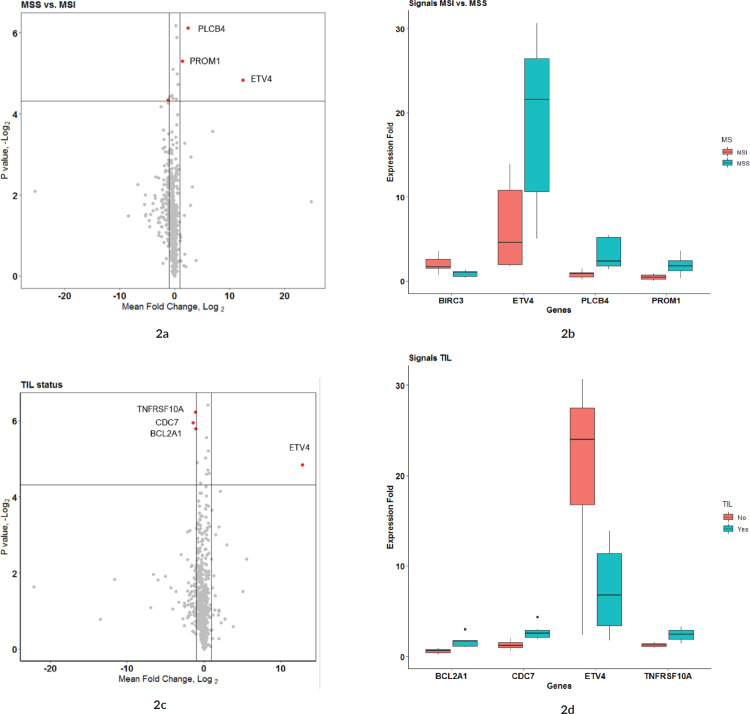
(a and b): Comparison of microsatellite instability status in gene expression patterns. (a): Group comparison of expression FC in MSI and MSS group using t-test. The X-axis shows the mean difference between the two groups for each of the signals and the Y-axis shows the −log_2_ (p-value). Horizontal intercept above 4 in the Y-axis is the line of significance, signals above which have a p-value < 0.05. The vertical line with X-intercept is at a log_2_(FC) of 1. There were only three signals with |log_2_(FC)| > 1 and p-value < 0.05. Four genes satisfied p-value < 0.05 and FC(log_2_) > 1 – ETV4, PLCB4, BIRC3 and PROM1 (marked by red dot). (b): This figure shows the expression FC in highly significant genes identified in Figure 2a. Among them, ETV4 has the highest expression in the MSS group compared to MSI. (c and d): Comparison of tumour immune cell infiltration status in gene expression pattern. (c): Group comparison of expression FC in TIL versus none using t-test. Axis annotations and intercepts are the same as that of Figure 2a. Four signals with |log_2_(FC)| > 1 and p-value < 0.05 were ETV4, TNFRSF10A, CDC7 and BCL2A1 (marked by red dots). ETV4 is the common signal in both these categories with maximum FC. (d): Boxplot depicting the expression FC in the four genes identified in Figure 4a. Among them, ETV4 is associated with high expression in the TIL ND group.

**Figure 3.**
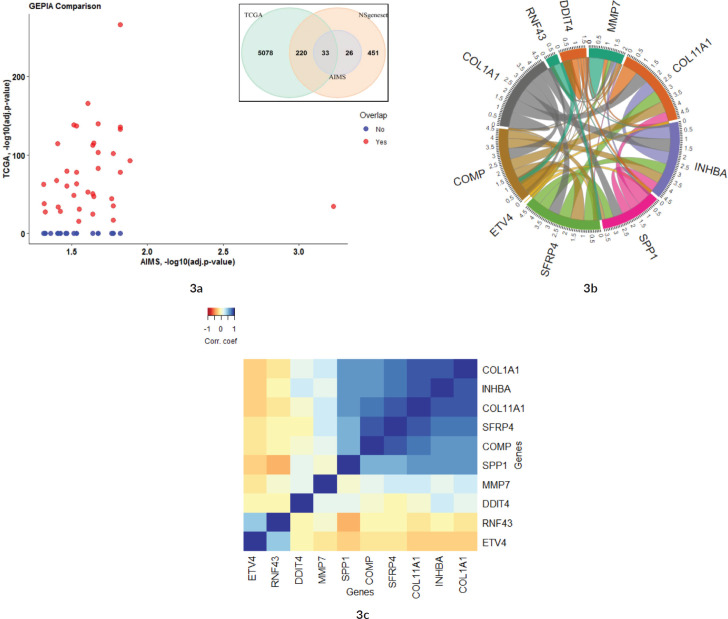
(a): Comparison of 59 significant genes in the current study correlated with TCGA COAD expression data analysed by GEPIA. The X-axis shows the p-values (−log_10_) of the current study and the Y-axis depicts the p-values (−log_10_) from the TCGA COAD data. 36 out of 59 genes had a significant difference in TCGA, while 23 genes were not significant in the TCGA data (as zero values in the graph). The Venn diagram shows the overlap of genes that are included in PanCancer pathway gene set of Nanostring and how much genes are overlapping in the two studies. (b and c): Co-expression correlation of top 10 upregulated genes in the study. (b): Chord diagram showing the correlation of top 10 genes that are correlated in the TIMER TCGA COAD database. The overall contribution of the correlation coefficient was found to be highest among six genes (COL11A1, COL1A1, COMP, SPP1, SFRP4 and INHBA; a score of 3.5–4.5). Genes that contributed less to overall correlation were DDIT4, RNF43 and ETV4 (score of 0.0–1.5). (c): Heatmap showing the co-expression of the top 10 genes in the TIMER database. Added to the high co-expression correlation with the six genes (COL11A1, COL1A1, SPP1, SFRP4, COMP and INHBA), there is a separate correlation between RNF43 and ETV4 in a different cluster. Details are described in the main body of the text.

To compare the effects of microsatellite instability (MSI) status and tumour immune cell infiltration (TIL) on the expression fold of mRNA signals, the expression FCs of all signals were compared separately against MSI status (MSS versus MSI groups) and TIL status (IHC detected or not in FFPE sections). The expression FC of 730 genes from the Nanostring DE analysis was compared between the MSI and MSS groups to reveal 13 genes that were significant (*p*-value < 0.05) (see Sheet 6 of Supplementary Dataset File S3). Among these, four genes had a mean |FC(log_2_)| > 1 between the two groups (Figure 2a). Of the four, ETV4 had the significant and highest FC (mean FC(log_2_) = 12.38; *p*-value = 0.035) in MSS group compared to MSI (Figure 2b). Other significant genes with FC > 1 were PLCB4 (mean FC(log_2_) = 2.43; *p*-value = 0.014), PROM1 (mean FC(log_2_) = 1.39; *p*-value = 0.025) and BIRC3 (mean FC(log_2_) = −1.13; *p*-value = 0.049). Expression of all these genes was significantly different in the TCGA as well, as shown in GEPIA comparative analysis (Figure 3a). When expression levels were compared against the TIL status, the only signal that was most significant with the highest mean FC was ETV4 (mean FC (log_2_) = 12.82; *p*-value = 0.035) (Figure 2c and d). Other signals that were significant between the groups, but FC(log_2_) ≈ 1 were BCL2A1 (mean FC(log_2_) = −1.061, *p*-value = 0.018), CDC7 (mean FC(log_2_) = −1.42, *p*-value = 0.016) and TNFRS10A (mean FC(log_2_) = −1.14, *p*-value = 0.013). In both these group comparisons, ETV4 was found to be the only signal that was conspicuously higher in the MSS group with no TIL. There was no significant difference in the gene expression pattern when compared against age, sex or anatomical location of the tumour.

### GEPIA correlation analysis

The expression profile in the current study Amrita Institute of Medical Sciences (AIMS) was compared against the TCGA COAD dataset from GEPIA. 36/59 significant genes in the AIMS study correlated with the TCGA COAD dataset (common set) in the GEPIA database (See Figure 3a and Supplementary Dataset File S4). However, 23 genes were found to be not significant in the GEPIA/TCGA dataset (unique set). The most significant gene in the TCGA from the AIMS dataset is ETV4, while overall in the AIMS study, HDAC4 was highly significant (see volcano plot in Figure 1a). Analysis of the top 18 of the 59 signals with |FC(log_2_)| > 2 revealed 14 genes from common set and four genes from unique set. The genes in common set, in the descending order of FC, were, MMP7, COL11A1, INHBA, SPP1, SFRP4, ETV4, COMP, RNF43, GZMB, MET, MCM2, LIF, KLF4 and FLNA. Among them, MMP7, ETV4 and RNF43 had an |FC(log_2_)| >5 in the GEPIA. Similar expression pattern (upregulation/down regulation) was shown by 34/36 common set genes in both data sets, while two genes (IL1RAP and HRAS) had a reversal in FC values. Among the 36 genes that were significant in the GEPIA/TCGA data, 24 are upregulated and 12 genes are downregulated in the current study.

### TIMER correlation analysis

The list of highly significant 59 genes obtained from the Nanostring was further analysed in the TIMER tool using TCGA COAD correlation module. Correlation module showed expression scatter plots between a pair of genes from the list in TCGA COAD cancer type with Spearman’s rho value with statistical significance. Correlation data is given in Supplementary Dataset File S5. As shown in the chord diagram, Figure 3b, expressions of COL1A1, COL11A1, COMP, INHBA and SFRP4 had a score of 4.5, and SPP1 had a score of 3.5, and all of them were found to be significantly correlated (Figure 3c). On the other hand, DDIT4, RNF43 and ETV4 had a low score (0.0–0.5) suggesting lower correlation. The correlation heatmap (Figure 3c) showed that ETV4 and RNF43 expressions were significantly correlated to each other.

### Network analysis

**Figure 4.**
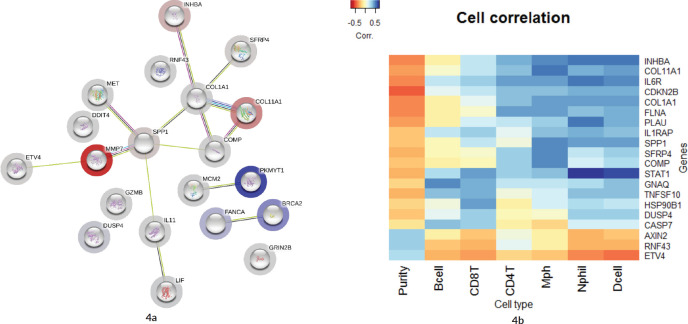
(a): Network by STRING.db of the top 20 FC genes that were identified from the expression analysis. All the interactions were highly significant (FDR adj. p-value < 0.01). All coloured nodes are the first shell of the interaction of each of the proteins. Edges represent protein–protein interaction, not necessarily connected. Colour codes of interaction lines represent gene neighbourhood (green), gene fusions (red), gene co-occurrence (dark blue), from curated databases (teal), experimentally determined (pink), text mining (yellow), co-expression (black), protein homology (light blue). The structures seen inside the nodes are the 3D structures of the known proteins. The colour of the halo around the shells depicts the rank of each protein based on the expression FC, whether upregulated (red) or downregulated (blue). (b): Comparison of the top 10 genes from the current study to immune cell infiltration in the TCGA TIMER database. Tissue macrophages (Mph) are highly associated with majority of the upregulated genes, followed by CD4-T lymphocytes, dendritic cells (Dcell), neutrophils, CD8-T lymphocytes and B-cells in the descending order. The tumour content (represented by ‘Purity’) is highly correlated with ETV4 and RNF43.

As illustrated in Figure 4a, 16 genes from the top 20 were found to be interacting with each other according to gene co-occurrence and gene-neighbourhood in STRING.db. Detailed list of interactions is given in Supplementary Dataset File S7. Among these, experimentally proven interactions were projected in four clusters. These were: 1) COL1A1 interacting INHBA, SFRP4, COL11A1, COMP and SPP1; 2) SPP1 with strong interaction with MMP7, which in turn interacts with ETV4, then MET, and weak interaction with IL1 which networks with LIF; 3) BRCA2 with FANCA and 4) PKMYT1 with MCM2 in separate interactions. Protein homology was found between COL1A1 and COL11A1, SFRP4 and WNT2, MMP7 and SPP1, suggesting homology-based interaction among these proteins. The correlation cluster is seen with the TIMER TCGA database, and the genes clustered in the STRING.db network analysis was corresponding to many genes with each other.

### Association of immune cell infiltration with gene expression

Figure 4b shows a hierarchical clustering analysis of the correlation among the top 20 genes, identified by the Nanostring analysis, with the immune cell infiltration data obtained from TIMER (Supplementary Dataset File S6). Six genes were associated with the immune cell infiltrates. COL11A1, COL1A1, INHBA, SPP1, SFRP4 and COMP were clustered with macrophages. Certain signals with high FC values, such as MMP7 and DDIT4, were not associated with any specific cell type. However, ETV4, RNF43 and AXIN2 were clustered with tumour purity and separate from all other infiltrating cell types.

## Discussion

### Approach to overlay gene expression study from different population on to TCGA

The molecular classifications specified by the TCGA are derived from tumour samples of a wide variety of tissues at various levels of tumour stages, infiltrating cellular types and different ethnic groups. Correlation of different data in TCGA to clinical medicine requires validation studies and analysis for their translational capacity in different populations. To that end, one approach would be to overlay annotated molecular signals from tumour samples from different populations on to the TCGA at multiple data layers and examine pathogenic role of individual signals in those populations. We have provided a set of methods based on this approach in the current pilot study of colon adenocarcinoma occurring in Kerala, a population that has not been widely represented in most of the previous large cohort studies, including TCGA. We, initially, compared the significant genes identified in the two datasets. Subsequently, the co-expression correlation between the genes from both datasets was determined and finally, the DE genes in the study cohort were compared with immune cell co-localisation in the TCGA data.

### ETV4 and MMP7 are significantly upregulated gene signals

Among all the DE genes in this cohort, in multiple group comparisons and against TCGA database by GEPIA and TIMER analysis, ETV4 was the most significant gene identified in this study cohort. This may be the first study to show a high correlation of ETV4 with MSS CRCs. ETV4 or ETS Variant Transcription factor 4 (E1A enhancer-binding protein – E1AF or Polyoma Enhancer Activator 3 Homologue) is a known transcription factor, upregulated and activated in colon adenocarcinoma [23–25]. The reduced expression of ETV4 in MSI tumours could be due to the presence of dinucleotide (GT) repeats in the third intron of the *ETV4* gene, causing frame-shift mutation in the coding region owing to its instability. In MSS tumours, ETV4, along with the *β*-catenin, induced *MMP7* in intestinal and colon cancer cells [26]. In the current study, ETV4 was found to be elevated in MSS samples as well as in subsection of MSI. The signals that induce the expression of ETV4 in MSS tumour cells are not yet fully understood. It is interesting to note that, ETV4, RNF43 and AXIN2 are highly correlated with tumour cell purity (Figure 4b). RNF43 and AXIN2 are negative regulators of the Wnt-ligand dependent type of CRC [27, 28] and their downregulation in MSI samples might suggest an association of Wnt ligand dependent pathogenesis in a subset of tumours.

### Gene clusters with cell types in tumour microenvironment

A cluster of genes that were upregulated in the tumours, compared to normal tissues were COL1A1, COL11A1, SPP1, COMP, SFRP4 and INHBA. Numerous studies have shown the proliferative role of collagen signals, COL1A1 and COL11A1, in gastric [29, 30], breast [31] and colon cancers [32–34]. MMPs (MMP7 and 9) along with collagen proteins were found to be important for tumour vascular invasion [35]. MMPs and signals of Epithelial–Mesenchymal Transition, COMP [36] and SFRP4 [37], were also found to be upregulated in colon cancers and associated with poor overall survival. Inhibin *β* A (INHBA) is a member of Transforming Growth Factor *β* superfamily, whose increased expression was shown in colon adenocarcinoma cells with prognostic significance [38]. The clustering of genes from the network analysis (Figure 3b and c) and TIMER correlation analysis (Figure 4b), in the current study, showed two distinct clusters of gene signals, residing in two different cell types/groups of the tumour. The proliferative role of these signals may apply to both tumour cells and infiltrating immune cells, as both are required for the tumour evolution and progression. Though the cell types associated with these signals were previously reported in several studies [39, 40], the signals correlated in the current study have not been associated with any of the tumour immune cells. The high-dimensional expression analysis in Nanostring assays is limited, in this context, to delineate the location of these signals in a complex tumour microenvironment. More elaborate studies such as IHC co-localisation experiments are required to identify the cellular location of these signals in tumours.

### Effect size and power analysis for future studies

We calculated the effect size for power analysis for future studies according to the method given previously(μ1–μ2/σ) [41]. From the normalised raw data, the effect size was found to be 1.414. With this effect size on a *t*-test, two-sided variation assumption, significance level of 0.05, power of 0.8 and the test sample size (*n*1) of 11, the normal sample size (*n*2) should have been 6. The number of normal samples included in the current study was lower than the calculated value. The overall variance of FC values of the normal set was found to be approximately eight times lower than the overall data variance of the tumour set. The effect size was found to be larger because of the uniformity of the samples taken for the study. All the samples were from Stage II CRC and stratified after histopathological evaluation and screening. This study aims to provide an approach to better utilise global datasets such as TCGA to understand the pathogenesis of CRC in local ethnic populations. The signals revealed in this study need to be further validated in larger patients’ samples in the same population in order to check pathogenic role and predictive and prognostic utility. Further studies should take into account the effect size and follow the selection of the samples as given in this study. We think methods like these, if implemented and confirmed in different populations in larger numbers, would help in the identification of more precise prognostic predictive markers and effective agents that can counter the pathogenesis to provide better patient care and effective intervention.

## Conclusions

We have shown, in this pilot study, a set of methods to bridge the gap between large cohort molecular pathogenesis discovery studies, such as TCGA, to low-throughput and uncharacterised tumour samples from routine clinical pathology laboratory, in an unique or highly focused ethnic population. We compared expression analysis using Nanostring PanCancer pathway panel in 11 Stage-II colon and rectal adenocarcinoma tumour from a population in Kerala, India, against TCGA COAD data. The signals revealed in the study were overlapped with TCGA at three different data layers such as correlation of different gene expressions to each other, correlation of gene expression to cellular co-localisation and correlation of gene expression to protein–protein interaction strength. Top 20 DE genes with largest FC were identified; two sets of gene signals were clustered, according to the comparative co-expression analysis using GEPIA and protein interaction network analysis. The effect size assessed from this pilot study could be adopted for further studies on larger number of samples in closely selected subsets of colon adenocarcinoma tumour samples.

## Conflicts of interest disclosure

The author(s) declare no competing interests.

## Author contributions

PSA has designed the experiments with RAJ, VM, RRP and KP. VM and DMV reviewed the project and further modified. Conduct of the research was done by PSA and RAJ. Data analysis was done by PSA. The manuscript was written by PSA and RAJ, reviewed by VM, RRP, KP and DMV.

## Data availability

Supplementary and other data are publicly available at the Open Science Forum website. The data can be accessed under ‘CRC Kerala Pathogenesis Project / Publication files’ from the weblink: https://osf.io/tcdk9/.

## Funding statement

The research was supported by an internal seed grant from Amrita Vishwa Vidyapeetham to PSA.

## Ethics committee approval statement

The study was conducted as per the guidelines of the Institutional Ethics Committee of Amrita Institute of Medical Sciences. The study was reviewed and approved by the IRB on 24 November 2017 (IRB-AIMS-2017-124).

## List of abbreviations

AIMS, Amrita Institute of Medical Sciences; Bcell, B Lymphocytes; CD4T, CD-4 T-lymphocytes; CD8T, CD-8 T-lymphocytes; COAD, Colorectal adenocarcinoma; CRC, Colorectal cancer/carcinoma; Dcell, Dendritic cell; DE, Differentially expressed; DNA, Deoxyribonucleic acid; FC, Fold-change; FDR, False discovery rate; FFPE, Formalin fixed paraffin embedded; GEPIA, Genome expression profiling interactive analysis; GTEx, Genotype-tissue expression project; IHC, Immunohistochemistry; MMR, Mismatch repair; Mph, Tissue macrophages; MSI, Microsatellite unstable; MSS, Microsatellite stable; Nphil, Neutrophil; PCA, Principal component analysis; PCR, Polymerase chain reaction; STRING.db, Protein-protein interaction network database; TCGA, The cancer genome atlas; TIMER, Tumour immune estimation resource; TNM, Tumour node metastasis Staging.

## Figures and Tables

**Table 1.** Patient and sample details.

ID	Age/sex	Location	MMR IHC	MSI-PCR	Lymphocyte infiltration	UICC tumour node metastasis staging group	Stage
KSN	38/F	Sig. colon	Normal	MSS	ND	T3N0Mx, LVI+, PNI−	IIB/IIIA
SIV	47/M	Ileo-cecum	Normal	MSS	Severe	T3N0Mx, LVI-, PNI−	IIB
KSM	52/M	Asc. colon	Deficient MLH1 & PMS2	MSI-H	Moderate	T3N0Mx LVI+, PNI−	IIB/IIIA
JCB	50/M	Asc. colon	Deficient MSH2 & MSH6	MSI-H	Moderate	T3N0Mx, LVI+, PNI−	IIB/IIIA
AN	46/M	Asc. colon	Deficient MSH2	MSI-H	Moderate	T3N0Mx, LVI+, PNI−	IIB/IIIA
ES	58/F	Tr. colon	Normal	MSS	Moderate	T3N0Mx, LVI+, PNI−	IIB/IIIA
SH	50/M	Asc. colon	Deficient MLH1 & PMS2	MSI-H	Moderate	T3N0Mx, LVI+, PNI−	IIB/IIIA
SK	54/M	Asc. colon	Normal	MSS	Moderate	T3N0Mx, LVI+, PNI−	IIB/IIIA
KNR	76/F	Tr. colon	Deficient MLH1 & PMS2	MSI-H	Mild	T3N0Mx, LVI+, PNI−	IIB/IIIA
VVC	67/M	Asc. colon	Deficient MSH2 & MSH6	MSI-H	ND	T3N0Mx, LVI-, PNI−	IIB/IIIA
RV	67/F	Asc. colon	Normal	MSS	Moderate	T3N0M0	IIB
